# Computerized Assessment of Psychosis Risk

**DOI:** 10.20900/jpbs.20210011

**Published:** 2021-06-29

**Authors:** Vijay A. Mittal, Lauren M. Ellman, Gregory P. Strauss, Elaine F. Walker, Philip R. Corlett, Jason Schiffman, Scott W. Woods, Albert R. Powers, Steven M. Silverstein, James A. Waltz, Richard Zinbarg, Shuo Chen, Trevor Williams, Joshua Kenney, James M. Gold

**Affiliations:** 1 Institutes for Policy Research (IPR) and Innovations in Developmental Sciences (DevSci), Departments of Psychology, Psychiatry, Medical Social Sciences, Northwestern University, Evanston, IL 60208, USA; 2 Department of Psychology, Temple University, Philadelphia, PA 19122, USA; 3 Departments of Psychology and Neuroscience, University of Georgia, Athens, GA 30602, USA; 4 Department of Psychology and Program in Neuroscience, Emory University, Atlanta, GA 30322, USA; 5 Department of Psychiatry, Yale University, New Haven, CT 06519, USA; 6 Department of Psychological Science, 4201 Social and Behavioral Sciences Gateway, University of California, Irvine, CA 92697, USA; 7 Center for Visual Science, Departments of Psychiatry, Neuroscience and Ophthalmology, University of Rochester Medical Center, Rochester, NY 14642, USA; 8 Maryland Psychiatric Research Center, Department of Psychiatry, University of Maryland School of Medicine, Baltimore, MD 21228, USA; 9 Department of Psychology, Northwestern University, Evanston, IL 60208, USA; 10 The Family Institute at Northwestern University, Evanston, IL 60208, USA

**Keywords:** clinical high-risk, psychosis, schizophrenia, prodrome, risk screening, behavioral tasks, computational psychiatry, precision medicine, computerized assessment, risk calculator

## Abstract

Early detection and intervention with young people at clinical high risk (CHR) for psychosis is critical for prevention efforts focused on altering the trajectory of psychosis. Early CHR research largely focused on validating clinical interviews for detecting at-risk individuals; however, this approach has limitations related to: (1) specificity (i.e., only 20% of CHR individuals convert to psychosis) and (2) the expertise and training needed to administer these interviews is limited. The purpose of our study is to develop the computerized assessment of psychosis risk (CAPR) battery, consisting of behavioral tasks that require minimal training to administer, can be administered online, and are tied to the neurobiological systems and computational mechanisms implicated in psychosis. The aims of our study are as follows: (1A) to develop a psychosis-risk calculator through the application of machine learning (ML) methods to the measures from the CAPR battery, (1B) evaluate group differences on the risk calculator score and test the hypothesis that the risk calculator score of the CHR group will differ from help-seeking and healthy controls, (1C) evaluate how baseline CAPR battery performance relates to symptomatic outcome two years later (i.e., conversion and symptomatic worsening). These aims will be explored in 500 CHR participants, 500 help-seeking individuals, and 500 healthy controls across the study sites. This project will provide a next-generation CHR battery, tied to illness mechanisms and powered by cutting-edge computational methods that can be used to facilitate the earliest possible detection of psychosis risk.

## INTRODUCTION

Schizophrenia (SZ) is among the top causes of disability. Despite successful management of positive symptoms in many cases, the majority of patients demonstrate significant disability over much of their adult lives as well as premature mortality [1,2]. Several potentially modifiable risk factors for poor outcomes have been identified, including longer duration of untreated psychosis (DUP) [3–6]. The present multi-site study aims to address this by facilitating cost-effective, brief, and broadly-available screening to promote early detection of elevated risk for onset of psychosis so that DUP can be minimized and future preventative intervention trials can conveniently and cost-effectively identify those at greatest risk.

Individuals showing newly-emergent or escalating attenuated positive symptoms (e.g., hearing sounds without identifiable source), and/or with a first-degree relative with a psychotic disorder coupled with a recent decline in functioning, are considered to be at clinical high-risk (CHR) for transition to psychosis [7,8]. The CHR period is a critical time for early intervention, and a number of specialty clinics have been established with the goal of delaying or preventing the onset of psychosis, and improving the course of illness in people who convert to psychosis. The first generation of CHR studies focused on the development of reliable clinical interview methods to identify young people who appeared to be at the highest risk for conversion to psychosis so that they could receive careful monitoring and treatment as appropriate [7–15]. This approach has substantially improved our understanding of the prodrome and highlighted biomarkers associated with CHR status and prediction [16–28]. One important product from this effort is the North American Prodromal Longitudinal Study (NAPLS) risk-calculator [17,18], which is a tool that shows favorable test characteristics (sensitivity and specificity well beyond chance, and beyond the clinical interview CHR diagnosis alone) in predicting who may eventually convert to psychosis. However, the data needed for the NAPLS calculator are based on specialized interviews and neuropsychological testing, requiring expertise that involves extensive training and is only available in a small number of academic clinical settings [8]. We believe in light of recent advances in clinical cognitive neuroscience and computational psychiatry that it is now possible to develop a new approach to the prediction of conversion to psychosis that builds upon the pioneering CHR work, but exploits discoveries in the cognitive neuroscience of psychosis that came after the initiation of NAPLS and similar projects. Focusing on neurocognitive mechanisms implicated in symptom formation and maintenance will facilitate the translation from prediction to prevention. By using simple computerized tasks that are strongly tied to cognitive and computational neuroscience models of specific symptom clusters, we will expedite the transition from the laboratory to real-world clinics.

### Problems with Current Approach

#### Low Specificity.

As noted, before a risk calculator can be applied, the diagnosis of a CHR syndrome is necessary. Indeed, all current approaches to CHR research and treatment rely on a specialized structured clinical interview, a method that has limited specificity. Only 15–30% of individuals who meet CHR criteria convert to psychosis over extended follow-up [7,21,29–36]. Low conversion rates found with current screening methods seriously confound attempts to power primary prevention intervention trials, as seen in the negative findings of the NEURAPRO fish oil study [37,38]. As one of the NIMH long-term strategic goals is to develop and test primary preventative interventions for psychotic disorders, there is a need to increase the predictive accuracy of assessment of imminent risk, in order to enrich samples for future treatment trials [39–41].

#### Limited Availability.

Current methods for CHR identification are based on interviews that require extensive training, in addition to the establishment of referral networks (relying on recruitment specialists and community-clinic training) or resource-intensive public health awareness campaigns. As a result, only a minority of young people who develop psychosis are ever diagnosed with, or access specialty care for CHR syndromes. Even in the UK, where specialty CHR care is available via the National Health Service [35,39,42–48], only 5% of people with first episode psychosis have had any contact with CHR services [9,34,49,50]. In the US, the situation is even worse: CHR services are only available in a few settings [7,51–53], and this has limited the public health impact of the first-generation studies. We believe a very different approach is needed to expand the availability of CHR screening.

### Addressing the Problems with the Current Approach

#### New Metrics.

We propose to address the above critical issues in several ways. First, to address Issue 1 (“Low Specificity”) we will assess the predictive power of objective performance-based (perceptual, cognitive, affective, motor functioning) measures that are related to symptom severity (i.e., to specific aspects of clinical state). Importantly, each of these measures has been previously related to the computational, cognitive, and neurobiological mechanisms involved in either positive, negative, or disorganized symptoms. To further improve specificity, we will also include a measure on which SZ patients perform normally, but where people with non-psychotic mood disorders (common baseline and follow-up diagnoses in CHR patients) evidence impairment (e.g., hedonic reactivity) [54–56].

We have chosen measures with strong track records of state-sensitivity and symptom-specificity, many tied to neurocomputational models of these symptoms. These include measures motivated by Bayesian predictive coding (positive symptoms), models that emphasize local or large-scale context-based coordination of cortical processing (disorganization symptoms), and models that emphasize the role of impairments in reward processing or response initiation (negative symptoms). By focusing on specific psychotic disorder-relevant neurocognitive computations for risk prediction, we believe that predictive accuracy for a psychotic disorder will be significantly improved over the current NAPLS risk calculator. The mechanisms most strongly predictive of conversion provide clear targets for future treatment development.

#### More Accessible Tools.

Our approach also has practical advantages pertaining to Issue 2, (“Limited Availability”): If our computerized approach is successful, collecting the data necessary for CHR risk prediction will not require extensive training, nor be challenged by issues of inter-rater reliability. Overcoming these issues has the potential to substantially increase the availability and reduce the cost of CHR evaluations. These are critical issues if state-of-the-art CHR evaluations with strong predictive validity are to be delivered in non-specialty-clinical settings. Predictive models need to be tested in real-world situations, effectively distinguishing CHR from other help-seeking populations in these contexts as well.

Here, we seek to demonstrate that measures of perceptual, cognitive, affective, and motor functioning offer sensitivity to conversion to psychosis that meets or exceeds what has been achieved in prior research. From a public health perspective, our efforts would still have significance even if our risk calculator is less sensitive than the NAPLS calculator, because our approach is designed to have a broader reach. We have prioritized computerized measures that could reach non-help-seeking individuals, which we see as critical for future population-based studies and effective real-world outreach.

### Big Picture Goals—The Immediate and Long-Term Benefits of a New Tool

Most individuals who meet CHR criteria have a path to treatment that does not involve specialty CHR clinics, even when such a clinic is locally available [35,39,43–48]. Many may initially be seen by a pediatrician (based on parental concern), or by a school psychologist or guidance counselor (based on teacher reports), or by a college counselor based on self-referral. In these cases, the onset of a serious psychiatric disorder is not typically a focus of staff expertise. The tools we propose to develop can be disseminated online for use by community clinicians. These tools might even be accessed by young people who have concerns about their mental health using their own personal electronic devices—an approach that was recently shown to be feasible in the UK [57,58]. The results of our risk calculator could inform decisions by young people, their families, and community clinicians regarding seeking information and care [39]. We see this potential expansion in the availability of screening for psychosis vulnerability, beyond the geographical boundaries of academic specialty centers, to be the critical future impact of the proposed work.

## INNOVATION

### Conceptual

At a conceptual level, we are proposing a fundamental re-orientation in approach to the question of how to select measures sensitive to near-term conversion to psychosis. The “first generation” of CHR studies primarily focused on measures that had been shown to be markers of risk from family and “high-risk” study designs [59–62]. That was a sensible decision at the time; these measures were reliably abnormal in ill patients and their first-degree relatives, suggesting that these measures were assessing fundamental aspects of illness risk. However, that approach also inevitably led to poor specificity. That is, deficits on risk markers, such as the Continuous Performance Test (CPT), are often found in people who never develop actual clinical illness [59]. By contrast, we focus on measures assessing computations that are also involved in hallucinations, delusions, disorganization, or negative symptoms. That is, our focus is not on sensitivity to the diathesis for schizophrenia; it is on behavioral measures that are assays of the mechanisms involved in symptom expression. By shifting the focus to symptom-specific state-linked probes, we expect to gain increased sensitivity to the pathophysiological changes active in people who are progressing towards a diagnosable psychotic disorder.

### Methodological

At a methodological level, we propose to focus on behavioral performance and self-report measures. While brain imaging and EEG measures are clearly of interest, it is our view that such measures will always be limited to specialized academic research centers. Further, these measures are costly to obtain and analyze, and it is nearly inconceivable that private or public payers will be willing to pay for such measures given that conversion rates are so low based on SIPS ascertainment. It is our strong view that only inexpensive behavioral measures have the potential to be implemented on a wide scale.

We also propose to include a control group comprising clinical help-seeking controls – participants who fall short of a CHR diagnosis and/or have a significant history of psychopathology. It is noteworthy that the extant neurocognitive CHR literature nearly always focuses on comparisons between CHR individuals and an ultra “healthy” control group. However, when working in the context of psychosis-risk identification, the challenge clinicians face is not to distinguish people who have no psychiatric problems from those with fairly severe psychopathology, but rather to distinguish CHR syndromes that are prodromal from mood and anxiety pathology, and other symptoms, that may look like CHR and are severe enough to lead people to seek care [63–65]. We therefore propose to evaluate the performance of our battery in a cohort of subjects who fall short of meeting full CHR criteria and typically have complex mood and anxiety symptoms (help- seeking controls) [13,52,66–68]. A measure that effectively distinguishes these cases from true CHR cases will have significant public health impact as it will limit false positives and allow clinicians to appropriately allocate limited treatment resources. However, only a handful of cognitive studies have used help-seeking controls, which limits meaningful generalization from this extant literature [69–72]. In order for our battery to be maximally useful, we need to enhance sensitivity and document specificity: this requires the use of help-seeking controls.

At the level of implementation, a fundamental motivation of this proposal is to develop a set of measures that can be delivered online so that prediction of risk for psychosis can be brought to any clinician with access to the Internet and a young client who they are concerned may be at CHR, or even directly to those clients themselves. We acknowledge that other biomarkers may be more informative about the pathophysiology of psychosis. We further acknowledge that it is fully possible that imaging approaches (such as positron emission tomography [PET] imaging of dopamine [DA] availability) may prove to be very powerful, sensitive, and specific measures of psychosis risk [73,74]. However, we are certain that such measures will never be widely available due to the expertise required and the cost involved. Thus, we propose a very different approach than others have taken: to focus on measures that are each linked mechanistically to symptom severity, and that can be delivered over the Internet and impact clinical care on a wide scale in real-world settings.

## PRELIMINARY STUDIES

### Task Markers for Positive Symptoms

Three of the tasks we discuss below assay abnormalities in predictive coding, a theoretical framework that bridges psychological and neural levels of explanation and lends itself to formal computational modeling of positive (psychotic) symptoms.

#### Kamin Blocking [75].

This task emphasizes the role of mismatches between expectation and experience (called prediction errors, or PEs) in belief formation [76]. This task implicates learning driven by aberrant prediction error signals as a critical mechanism in delusion formation. In our food-allergy causal belief learning task (published in more than 10 papers, spanning health and illness), participants are asked to imagine that they are allergists and to learn the causes of allergic reactions in a fictitious patient [76]. On each trial, they are shown a meal consisting of one or two different foods that the patient had eaten. They are then given feedback regarding whether that meal caused an allergy. Their task is to learn to predict the outcome of each meal. Prior learning that one food (i.e., bananas) causes the allergy (across 10 consistent repetitions) prevents (blocks) learning that another novel food (i.e., mushrooms) could also cause an allergy (6 trials) [77]. In other words, no PE is generated because the outcome is fully predicted by the banana; hence, no learning occurs. On later trials, when participants receive feedback that mushrooms cause allergy (6 repetitions), a PE brain response is observed [77]. In our imaging work, aberrant PE correlates specifically with delusions (delusion-related distress, in particular, as measured with the Peters Delusion Inventory, PDI [78]. Based on our prior work, we predict CHR participants will exhibit weaker blocking. Those at clinical high risk will learn an inappropriate association between the blocked cue and allergy and learned it more strongly, expressing that belief with higher confidence.

#### Sine Wave Speech Task [79].

**Figure 1.**
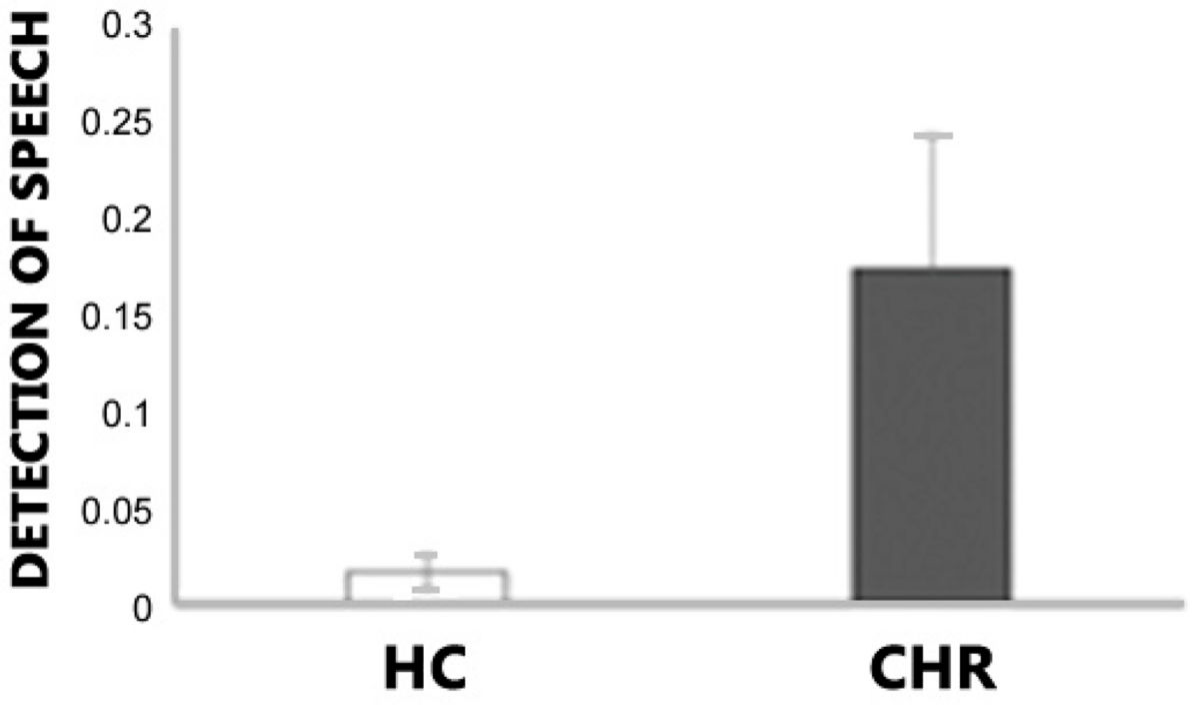
Detection of Speech in SWS. CHR detect more speech in SWS than controls.

This task provides a measure of the degree to which overweighting of prior beliefs (about speech) impacts sensory processing as a mechanism of hallucinations. Sine wave speech (SWS) is made by replacing the formants (main bands of energy) in speech with pure tone whistles. It is typically unintelligible on first exposure and may not even be recognized as speech. Once the listener knows that it is potentially intelligible as speech (by exposure to the pre-degradation speech template, which thus serves as a prior expectation), relatively high levels of comprehension are achieved. Individuals who hallucinate are able to perceive the speech in SWS, even before exposure to the pre-degradation speech template consistent with the presence of a strong prior for speech in people who hallucinate. In a paradigm adapted from Alderson-Day and colleagues [79] our subjects will passively listen to intelligible and unintelligible SWS. In Run 1, to disguise the presence of speech, subjects will be instructed to listen for a target cue (an equivalent noise-coded, unintelligible SWS stimulus, which sounds ‘noisier’/’rougher’), and told that the other sounds (unintelligible SWS) are ‘distractor’ stimuli. After Run 1, subjects will be asked if they noticed any words in the distractor stimuli. Hallucinating subjects report hearing speech in Run 1 and correctly identify more words in Run 1 compared to controls. Subjects are then explicitly told that there is actual speech in some of the stimuli (the ‘reveal’), and they will be exposed to some pre-degradation speech templates, and the task will be repeated (Run 2). We then test the ability of subjects to discriminate between intelligible SWS and unintelligible SWS (*d’*), their bias in classifying speech and non-speech, and accuracy (number of keywords correct). Prior work revealed no difference in d’ or bias on speech detection in Run 2. We predict that CHR converters (more than non-converters, HSC, and HC) will detect the speech in the degraded signal before the presence of speech is revealed in Run 1, but there will be no difference in Run 2. Importantly, this pattern of supranormal performance cannot be explained by generalized impairment, lack of effort, etc. Our preliminary data (Figure 1) support this prediction: CHR participants (*N* = 15) detected speech more readily in the sine wave stimuli than HCs (*N* = 17, *t* = 2.48, *p* = 0.019). This effect correlated significantly with the severity of SIPS positive symptoms (*r* = 0.37, *p* = 0.039) and hallucinations specifically (SIPS perceptual domain P4), at a trend level, in this preliminary sample (*r* = 0.33, *p* = 0.065).

#### Conditioned Hallucinations Task (CHT).

This task provides a measure of the degree to which subjects overweigh prior beliefs in sensory processing, a potential mechanism of hallucinations. The task engages Pavlovian conditioning to experimentally engender hallucinations [80]. Subjects undergo a test of auditory thresholds and then perform a conditioning paradigm (12 blocks of 30 trials), during which they see a visual checkerboard paired with a 1KHz tone stimulus at 75%, 50%, 25% or 0% (no tone) of their detection threshold. On each trial, they press a button to indicate whether or not they heard a tone and hold the button down for longer to express their confidence in that decision. Across task blocks, the checkerboard-tone association is degraded such that more and more no-tone trials are presented. Participants with and without hallucinations were recruited. After conditioning, all participants confidently reported hearing some tones that had not been presented (i.e., conditioned hallucinations). However, participants with a history of clinically-significant hallucinations reported conditioned hallucinations at a much higher rate. We next employed a formal computational model of perception that considers perceptual beliefs and incoming sensory input to model participant responses: a three-tiered Hierarchical Gaussian Filter (HGF). Consistent with the predictive coding account [81], we found that those with hallucinations demonstrate an over-reliance on prior beliefs. We predict that susceptibility to develop conditioned hallucinations and failure to update perceptual beliefs will be predictive of conversion to psychosis. In a preliminary data set, CHR participants showed an increased rate of conditioned hallucinations relative to healthy controls.

### Task Markers for Disorganization Symptoms

Experimental and computational studies indicate that disorganization reflects fragmentation in the coherence, or context-based linking, of mental representations, and in the sequencing of thought and motor behavior [82]. There is replicated evidence that reduced perceptual organization is associated with greater formal thought disorder and overall levels of disorganization symptoms [82–84]. Here we include two tasks that test the idea that reduced contextual modulation (thought to depend on connectivity within and between cortical regions) contributes critically to disorganized thought and behavior.

#### Ebbinghaus Illusion Task.

**Figure 2.**
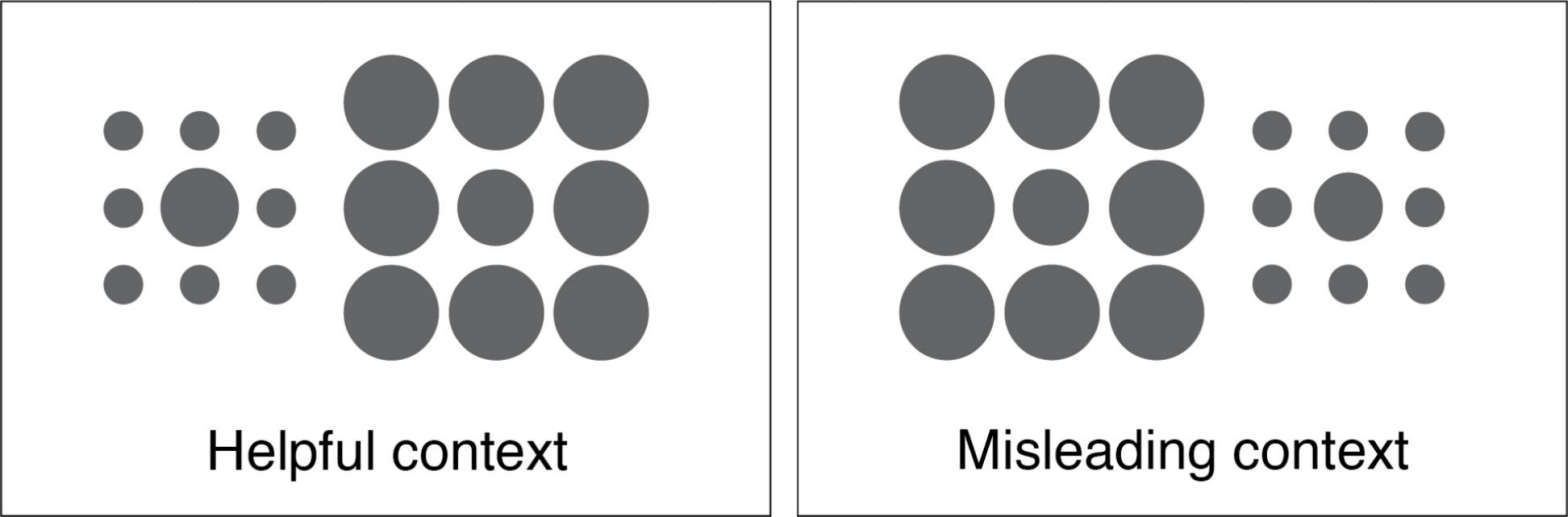
Ebbinghaus illusion example.

Reduced susceptibility to this illusion (see Figure 2), a marker of impaired visual context processing, is believed to arise due to reduced grouping of target and contextual stimuli [85]—a process tightly coupled with active disease processes; indeed, our team has observed that such abnormalities are present in active states of psychosis but then normalize as persons with psychosis remit [86]. Mittal, Silverstein, and colleagues evaluated 33 CHR and 40 controls with the same computerized version of the Ebbinghaus task used in prior studies by Silverstein and colleagues [86–91]. Participants were asked to judge which of two target circles is larger. The two target circles appeared simultaneously on the screen, either by themselves (no-context condition), or within a context that made size judgment easier (helpful condition in which surrounding the larger of the two inner circles by small circles normally creates the illusion that that inner circle is larger than its true size) or more difficult (misleading condition in which surrounding the smaller of the two inner circles by large circles normally creates the illusion that that inner circle is smaller than its true size). Susceptibility to this illusion (reflective of normative function) is measured as the difference between: (1) accuracy in the helpful condition and accuracy in size-difference-matched no-context trials (i.e., helpful index), and (2) the absolute value of the difference between accuracy in the misleading condition and accuracy in size-difference-matched no-context trials (i.e., misleading index). As predicted, both groups exhibited approximately the same percentage of accurate responses in the no-context (control) condition, and critically, there was a significant group-by-condition interaction (*F*(1,71) = 4.00, *p* ≤ 0.05) in which the CHR group (*M* = −44.46%; *SD* = 26.53%) was significantly more accurate than controls (*M* = −53.63%, *SD* = 12.98%), *t*(71) = 1.82, *p ≤* 0.05) on the misleading-index. Lower scores on the misleading-index (i.e., less susceptibility to the illusion and therefore more accurate size perception) were associated with increased disorganization (*r* = 0.34, *p ≤* 0.01) while a correlation for the helpful-index did not approach significance. These results indicate that visual context processing is impaired in CHR, and is linked to the severity of disorganization, as it is in first-episode and chronic SZ samples [86–90,92].

#### Mooney Faces Test.

This test involves perception of degraded pictures of human faces where all shades of gray are removed, leaving all features rendered in black or white only. On each trial the subject has to respond simply whether they do or do not perceive a face in the image. Perception of Mooney faces involves the grouping of the fragmentary parts into coherent images based on the perceptual organization principle of closure. Our original version of the task used 43 different face stimuli. In the ‘upright’ condition, the 43 faces are presented in their normal orientation. In the ‘inverted’ condition, the 43 faces are presented upside down, which significantly decreases the likelihood of perceiving a face. We previously demonstrated that reduced performance on this test is related to increased levels of disorganized symptoms in SZ [84,89] and others have demonstrated a relationship between reduced face perception in the upright (but not inverted) condition and disorganized symptoms [93,94]. In preparation for the grant resubmission, we collected data on 37 CHR subjects and 29 matched healthy controls. We observed that the CHR group was more likely to perceive a face in both the upright (*p* < 0.001, Cohen’s *d* = 0.89) and inverted (*p* = 0.055, *d* = 0.49) stimulus conditions than controls [95]. While this runs counter to our original hypothesis, it raises the intriguing possibility that the data reflect an excessive reliance on priors in the CHR group, (which is consistent with our preliminary data on the conditioned hallucinations and sine wave speech tasks). This hypothesis was supported by an additional finding from the study, that extent of reporting faces was significantly related to higher SIPS ratings on the perceptual distortions item (although this only occurred for male CHR subjects). We have decided to retain this task in the CAPR battery as a larger sample is needed to determine if the task is sensitive to disorganization or positive symptoms, or both, in this population. In the ongoing study, we have refined the task so that we are including a set of scrambled images that can serve as a noise condition, allowing for signal detection analyses. We are also asking subjects to respond on each trial for which they report a face whether the face is of a child or adult, or a male or female, to further assist with isolation of perceptual sensitivity and response bias.

### Task Markers for Negative Symptoms

There is consistent evidence that negative symptoms are associated with deficits in multiple aspects of reward processing and response initiation (e.g., reinforcement learning, effort-cost computation, value representation) that are needed to guide decision-making and motivate action [96]. Our preliminary data indicate that these same reward-processing abnormalities are present in CHR youth and predict greater negative symptom severity.

#### Pessiglione Reinforcement Learning (RL) Task [97].

This task tests the hypothesized role of impaired representation of expected value in guiding learning as a critical mechanism of avolition. The Pessiglione task is a measure of reinforcement learning that examines learning from gains versus losses [97]. There are 160 learning trials where 4 stimulus pairs are presented in an interleaved fashion, with participants receiving probabilistically reinforced feedback based on their choices. In two of the stimulus pairs, the correct choice leads to a monetary reward on either 90% or 80% of trials, with incorrect choices leading to a failure to make money; in the other two pairs, the correct choice leads to the avoidance of a monetary loss on 90% or 80% of trials. On the Pessiglione RL task, people with SZ display impairment in learning from gains, but intact learning from losses; poor learning from gains also predicts greater negative symptom severity. Preliminary data on the Pessiglione collected in Dr. Strauss’ lab indicates that CHR youth also have a deficit in learning from gains, but intact learning from losses compared to controls. As in SZ, greater negative symptom severity correlates with poorer learning from gains in CHR youth.

#### Effort Expenditure for Rewards Task (EEfRT) [98].

This task provides a measure of the degree to which the over-estimation of the cost of effort may be a critical mechanism in negative symptoms. Multiple studies indicate that SZ patients display a reduced willingness to exert higher levels of effort in exchange for increasing rewards [99,100], and that reduced effort is associated with greater negative symptom severity [101]. The EEfRT is used to measure effort-cost computation; it requires participants to choose between performing a low effort task (30 button presses within 7 s with the dominant hand index finger) for a lower reward value ($1) versus a high effort option (100 button presses within 21 seconds with the nondominant hand little finger) for higher reward values ($1.24–$4.30). Probability of reward receipt is manipulated across trials with cues at the start of each trial indicating a high (88%), medium (50%), or low (12%) probability of receiving money on that trial. The key dependent variable is the rate of selecting the high effort choice across probability and magnitude levels. Similar to what is observed in individuals with SZ, published data from Dr. Strauss’ lab indicates that CHR youth are also less willing to exert high effort to earn monetary rewards compared to controls, and that reduced effort is also associated with greater negative symptom severity [102].

#### Delay Discounting [103].

This task provides a measure of the degree to which the value of future rewards are discounted, a potential mechanism underlying motivational impairments [96]. On the delay discounting task, participants select between receiving smaller immediate rewards vs larger delayed rewards, SZ patients have been shown to prefer smaller immediate rewards over larger delayed rewards [104]. Furthermore, greater preference for smaller immediate rewards has been associated with greater severity of negative symptoms [98,105,106]. Published results from Dr. Strauss’ lab indicate that CHR youth also display delay discounting abnormalities compared to controls. These deficits reflect a failure to systematically increase preference for delayed rewards as value shifts from medium to large incentives. Furthermore, in CHR, failure to represent the value of larger future rewards as reflected by steeper discounting rates is associated with greater negative symptom severity.

#### Finger Tapping [107].

This task provides a measure of the ability to initiate volitional movements. The Computerized Finger Tapping Test (CTAP) measures how quickly the participant can press the spacebar using their index finger [106]. The test presents five, 10-second trials for the dominant hand alternating with five trials for the non-dominant hand 10s, cued by presentation of the green “GO” screen. Volitional movement is further assessed in the Variable Tapping and Tempo Tapping tasks by asking participants to match the pace of a series of tones when they tap the spacebar using the index finger of their dominant hand. In a preliminary study, examining a variant of the speeded condition alone, a sample of 41 CHR and 32 controls, CHR subject demonstrated significant slowing (*p* = 0.03) relative to controls and, tapping performance correlated specifically with negative symptom severity, *r* = 0.37, *p* = 0.03.

#### Hedonic Reactivity Task [56].

Numerous studies indicate that SZ patients demonstrate normal hedonic responses when exposed to pleasant stimuli [108], with individual differences in hedonic response being correlated with clinically-rated anhedonia (*r* = −0.51, *p* < 0.01).110 Data from Dr. Strauss’ lab indicates a different pattern in CHR youth, who were asked to make unipolar reports of positive or negative emotion, and arousal in response to pleasant, unpleasant, and neutral scenes from the International Affective Picture System (IAPS) [109]. CHR youth reported less positive emotion to pleasant stimuli than controls [56]. Furthermore, less positive emotion was associated with greater severity of anhedonia and mood disorder diagnosis accounted for 8% of variance in hedonic response. Analogous results were also found by a study from Dr. Mittal’s lab that used a similar task [110]. These findings suggest that unlike SZ patients, who exhibit intact hedonic responsivity at the group level, CHR youth display diminished hedonic capacity that is driven by depression. This is consistent with evidence that the hedonic response mechanism is intact in SZ, but impaired in mood disorders. Thus, we expect that normal performance on this task will be related to later conversion, whereas reduced hedonic response will predict non-conversion and likelihood of a mood disorder diagnosis. Thus, we anticipate that this measure may contribute to the risk calculator by offering negative predictive power.

## RESEARCH DESIGN AND METHODS

### Overview

**Figure 3.**
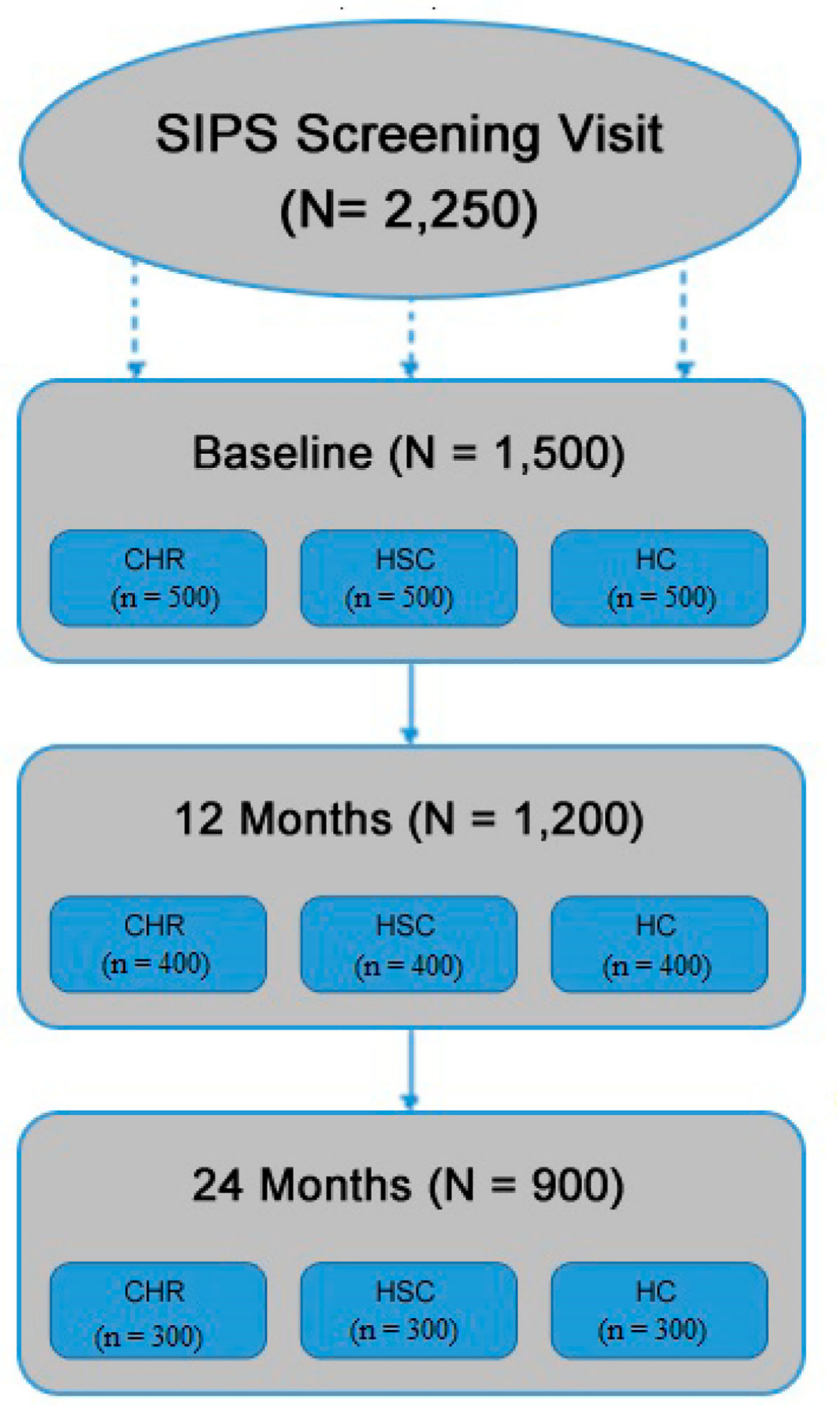
The recruitment flow and expected sample sizes across all study time points. Sample sizes are for the collaborative project and will split equally across the 5 sites. To account for possible attrition, we will continue to recruit until we have reached 1500 baseline interviews. Note. Abbreviations: Clinical high risk (CHR); help-seeking controls (HSC); healthy controls (HC).

The present multi-site study was funded in April of 2020 by the National Institute of Mental Health and data collection commenced in late 2020. Primary study sites include: Northwestern University, University of Maryland-Baltimore County, Yale University, University of Georgia, and Temple University. In addition, subcontracted sites, actively collecting data, include Emory University and the University of California Irvine. Due to the COVID-19 pandemic, and related safety and social distancing policies, it was necessary to begin the study remotely. Thus, the methods for the project were adapted so that all screening, baseline, and follow-up sessions will be conducted via Zoom or Webex (i.e., HIPAA-compliant secure videochat platforms) and all behavioral tasks will be implemented over the internet. An online platform for task implementation was built to accommodate remote administration. Although remote, each participant is guided through tasks by live research assistants, supervising the sessions. When the policies around in-person interaction return to prepandemic standards, the administration of the interviews and task battery will remain computerized, in an effort to standardize the experience for the participants. However, participants will have the option of participating at remote locations, or in the laboratory of one of the CAPR study sites. A total of 1500 participants will be recruited (500 CHR, 500 HSC, 500 HC), with recruitment divided evenly across the five sites (300 total per site: 100 CHR, 100 HSC, 100 HC). In addition, participants completing baseline assessments in Years 1–3 will return for 12 and 24-month visits, and participants completing baseline assessments in Year 4 will return for 12-month follow-up visits as well. See Figure 3 for a summary.

Each potential CHR participant will attend a 1.5-h screening session (i.e., Demographics and SIPS screening interview) and then be classified either as CHR (those meeting criteria for a progressive psychosis-risk syndrome) or control. All participants will attend a baseline session (4.5 h) consisting of: (1) a clinical assessment battery (remainder of SIPS, SCID) including a socio-occupational functioning interview and self-report measures; and (2) the computerized assessment of psychosis risk (CAPR) battery, as well as (3) tasks necessary to complete the NAPLS risk calculator and (4) a battery of self-report instruments. Following the baseline, control participants will be classified as a help-seeking control (HSC) or healthy control (HC), based on SCID diagnoses. Each follow-up session will take 2 h, and consist of SIPS, NSI-PR, SCID and socio-occupational interviews. This burden is consistent with prior CHR studies, and we have instituted a number of strategies to ensure tolerability.

### Participants

A total of 1500 participants, ages 12–34 will be recruited over a 5-year period across the collaborating sites. The upper age limit of 34 years was chosen as this includes the adolescent and young adult populations of interest [8]. Subjects in the CHR group will meet progressive or persistent psychosis-risk syndrome criteria on the basis of the SIPS interview and/or APS criteria on the basis of DSM-5. The HSC participants will include those who were referred or self-referred for a psychosis risk interview, but did not meet formal criteria for any psychosis-risk syndrome on the SIPS or APS criteria in the DSM-5 (note: these individuals may also have a family history of psychosis, but will not show the accompanying functional decline necessary for a formal psychosis risk syndrome diagnosis). In addition, participants that were initially recruited for the HC group, but observed to meet current or past SCID diagnoses will be included as HSC participants (note: past history of mild substance use will be allowed in the HC group). HC will include individuals with no family history of psychosis, or past/current serious psychopathology (e.g., psychosis, bipolar disorder, substance use disorder). Note: in service of external validity, we will recruit HCs exhibiting normative variation in anxiety and depression, but not taking psychotropic medication, consistent with NAPLS inclusion criteria.

#### Comorbidity.

CHR participants and HSCs are expected to present with comorbid diagnoses, most commonly depression and social anxiety [111,112]. We will carefully assess and monitor all comorbid diagnoses, both categorically and continuously, and include this information in our statistical models.

#### Substance Use.

Substance use disorder and evidence dependence *(*i.e., the participant shows tolerance for a substance, experiences withdrawal symptoms, and shows continued use despite significant impairment caused by taking the substance) is an exclusionary criteria and the participant will be asked about any history of drug dependence during the screening. If the participant endorses drug dependence within the past 6 months, they will be excluded. However, across all groups, we will include subjects with a history of substance use disorders (as noted, past mild substance use disorder history will be allowed in the HC, whereas the full range of possible severity of past substance use will be allowed for the HSC and CHR groups) as excluding them would lead to unrepresentative patient samples [7,113]. Substance use will be carefully monitored throughout the study.

#### Medication.

To maximize external validity, we will include CHR and HSC participants with current and past treatment with antipsychotic, antidepressant, and anxiolytic medications, as there is a growing trend to use these medications in youth [114,115]. Further, participants may choose to seek treatment during the course of the study (and this will not be grounds for exclusion). Instead, to promote external validity, we will carefully monitor medication and model influence. We will employ the manualized strategy used in NAPLS, recording for each medication course, start date, stop date, medication name and code, daily dose, and adherence (0 to 100%). Co-PI Woods, an expert in this area, will oversee data quality and lead monthly team consensus calls.

#### Recruitment and Feasibility.

The recruitment infrastructure is in place and each site is well situated to achieve the target goal of *N* = 300 per site. For instance, in recent years, all sites have recruited on average over 20 CHR participants per year, which will be sufficient for the present study. A variety of recruitment procedures will be used, including: print advertisements, campus postings, and bus and train advertisements, electronic advertisements, mail-outs to community health care providers, radio advertisements, and potentially other methods as well.

#### Attrition.

Our recruitment goals and power estimates account for estimated attrition and data loss. Based on our prior studies, we conservatively estimate that 15% of subjects will need to be excluded due to data loss and attrition over the course of 24 months. Thus, 390 will be recruited to reach the target *N* of 300 per site.

### Measures-Interviews and Clinician Ratings

Trained interviewers will gather a variety of data from participants, ranging from structured clinical interviews to observational data. In addition to the interviews listed below, interviewers will gather information on demographic, traumatic brain injuries, developmental history, medical concerns, and psychiatric history. All interviewers will complete intensive training on structured interviews and assessments (e.g., multi-day workshops), including close supervision of initial assessments with participants. Each individual site has a clinical psychologist with expertise in psychosis risk and thus will provide close ongoing supervision. In addition to this, a weekly clinical consensus meeting will be conducted to confirm SIPS ratings and diagnoses, to ensure that the instrument is used uniformly across all sites. Reliability will be assessed by randomly selecting 10% of interviews across the sites and coding interviews based on video recordings every 6 months by study interviewers. Kappa and ICC scores of 0.80 or higher will be judged reliable. If scores fall below 0.80, discrepancies will be examined and discussed among the PIs and all study interviewers to address potential drift and site differences.

#### Structured Interview for Psychosis-Risk Syndromes (SIPS), Version 5.6.

The SIPS is the most commonly used interview in the US for assessing psychosis-risk syndromes and has established predictive validity for conversion to psychosis, specificity, and inter-rater reliability [8,10,116]. Participants will be deemed at CHR for psychosis if they meet criteria for one or more (of 3) of the primary SIPS psychosis-risk syndromes at a progressive (recently emergent or escalating) or persistent designation. We also will examine the DSM-5 attenuated psychosis syndrome (assessed through the SIPS) and alternate SIPS 5.6 risk syndromes (e.g., persistence) in supplementary analyses. HSC and HC will not meet criteria for psychosis-risk syndromes.

#### The Structured Clinical Interview for DSM-5, Research Version (SCID).

Presence of *DSM-5* diagnoses will be determined using the SCID. Conversion to psychosis will reflect the presence of a *DSM-5* Schizophrenia Spectrum disorder (including schizophrenia, schizophreniform disorder, and brief psychotic disorder), or affective psychosis (including depression and bipolar disorder with psychotic features). These disorders reflect the standard for CHR research [12,117,118]. Additionally, the SCID will be used to identify comorbid diagnoses and differential HC and HSC participants.

#### Global Functioning Scale: Social and Role (GFS-S/R).

Social functioning will be assessed with the GFS-S [119], which provides ratings on a 10-point Likert scale. A score of 10 reflects “Superior Social/Interpersonal Functioning” (e.g., frequently seeks out others and has multiple satisfying interpersonal relationships including close and casual friends), whereas a score of 1 indicates “Extreme Social Isolation” (e.g., no social or family member contact at all). On the GFS-R, a score of 10 indicates “Superior Role Functioning”, whereas a low score of 1 reflects “Extreme Role Dysfunction”. Both the GFS-R and GFS-S were developed for CHR studies and have been found to be valid and reliable [7,119–121].

#### Negative Symptoms Inventory-Psychosis Risk (NSI-PR) [122,123].

The NSI-PR is a semi-structured interview that is used to rate 11 items anchored on a 0 (absent) to 5 (extremely severe) scale. The 11 items measure the 5 domains identified in the NIMH consensus conference: anhedonia, avolition, asociality, blunted affect, and alogia.

#### Family Interview for Genetic Studies (FIGS).

Participants will answer questions about symptoms, diagnoses, hospitalization, suicide, and alcohol and drug use in family members in a semi-structured interview [124]. In addition, a questionnaire based on the screening questions of the FIGS was developed by Dr. Ellman (Co-PI) as a guide for gathering diagnostic information about relatives in the pedigrees being studied in a brief online format that also is being administered in order to have future iterations of the battery that do not require interviewers.

#### Medication Log.

During the clinical interview portion of the study, assessors will use the medication log to collect information on participants’ medication history and usage, including treatment start and stop dates, medication dosage and type, and compliance.

#### Childhood Trauma and Abuse Scale (CTAS) [125].

Trained assessors will ask participants about history of trauma and abuse in 6 domains: psychological bullying, physical bullying, emotional neglect, physical abuse, psychological abuse, and sexual abuse. Assessors will not ask follow up questions and will only ask which trauma types have occurred in the lifespan.

#### Life Events Checklist.

Assessor will guide participants through a checklist of stressful events that have occurred in their lifetime [126]. Assessors will ask about the number of incidents and stress level of each endorsed item out of a 1–7 scale.

### Measures-Neuropsychological

#### Wide Range Achievement Test (WRAT) [127].

Participants will be shown a sheet with words listed on it ranging from simple to difficult. They will be asked to read the words and the assessor will keep track of incorrectly pronounced words. This assessment has been used as a reliable measure of general intelligence. Typically, general intelligence tests take several hours, and this is a quick and easy way to get a proxy of this information.

#### Brief Assessment of Cognition in Schizophrenia-Symbol Coding (BACS) [128].

The BACS assesses the aspects of cognition found to be most impaired and most strongly correlated with outcomes in patients with schizophrenia. In this study, we will be administering only the symbol coding component. The symbol coding task sheet will be mailed to participants in advance along with the headphones used for the computerized tasks.

#### Hopkins Verbal Learning Test-Revised (HVLT-R) [129].

The HVLT-R consists of a list of 12 nouns (targets) with four words drawn from each of three semantic categories. The semantic categories differ across the six forms, but the forms are very similar in their psychometric properties. Raw scores are derived for Total Recall, Delayed Recall, Retention (% retained), and a Recognition Discrimination Index. The purpose of this task is to assess verbal learning and memory within brain-disordered populations.

### Measures-NAPLS Risk Calculator

We will gather NAPLS risk calculator variables: age, sex, SIPS positive symptom items P1 and P2, cognitive scores from the digit symbol coding subtest of the BACS and Hopkins Verbal learning Test (trials 1–3), stressful life events from the Research Interview Life Events Scale, trauma from the Childhood Trauma and Abuse Scale, family history of psychosis from the Family Interview for Genetic Studies (FIGS), and decline in social functioning on the GFS-S [119,124,125,128,129].

### Measures-Computerized Assessment of Psychosis Risk (CAPR) Battery

All CAPR tasks are listed in Table 1, organized by the Positive (4 tasks), Negative (5 tasks), and Disorganized (2 tasks) symptom domains. With the exception of the Probabilistic Reversal Learning Task (see below), all tasks have detailed descriptions in the Preliminary studies section above and are not revisited here. At baseline, the standard versions of Pessiglione, Probabilistic Reversal Learning, and EEfRT tasks that offer monetary incentives will be administered to half the participants; the other half will receive a version using points as incentives. This will be important for translating the task to an online platform, where monetary incentives will not be possible. All other tasks will be administered identically to all participants.

#### Probabilistic Reversal Learning Task.

This task, a three-option probabilistic learning task, wherein participants learn and update reward associations in light of variable outcomes, due to anticipated but uncertain changes in reward between options (reversal events, expected volatility), and unanticipated changes in the underlying probabilities themselves (contingency transition, unexpected volatility), challenges participants to form and update beliefs about the value of each option and the volatility of the task environment. Participants choose between three decks of cards with hidden reward probabilities, selecting a deck on each turn and receiving positive or negative feedback (+100 or −50 points, respectively). They are instructed to find the best deck with the caveat that the best deck may change. Undisclosed to participants, reward probabilities switch among decks after selection of the highest probability option in nine out of ten consecutive trials (“reversal events”). Reward contingencies change from 90%, 50%, and 10% chance of reward to 80%, 40%, and 20% between the first and second halves of the task (“contingency transition”; block 1 = 80 trials, 90–50-10%; block 2 = 80 trials, 80–40-20%). Thus, there is expected volatility (reversal events) and unexpected volatility (contingency transitions) associated with the task, about which participants needed to form and update beliefs in order to perform the task.

### Measures-Self-Reported and Clinical History Information

Participants will fill out a battery of questionnaires using the online survey platform Qualtrics. These measures will allow us to examine the potential for self-report measures, easily administered over the Web, to enhance the predictive accuracy of the CAPR battery.

#### Prodromal States Questionnaire (PQB).

To determine the presence of self-report symptoms of psychosis-risk, the PQB [130] will be administered at baseline and follow-up time points. The inventory includes 21 items designed to assess symptoms of unusual thought content, suspiciousness, grandiosity, perceptual abnormalities, and disorganized communication on a 5 point Likert scale.

#### Motivation and Pleasure Scale-Self-Report (MAP-SR).

The MAP-SR [131] is a 15-item measure that utilizes a 5-point Likert scale to examine consummatory and anticipatory pleasure in social, recreational, or work domains; feelings and motivations to be around family, friends, and romantic partners; and motivation to engage in activities. The MAP-SR has been shown to have excellent internal consistency, good convergent validity, and relates consistently with measures of social closeness and role functioning.

#### Perceived Stress Scale (PSS).

The PSS [132] measure consists of 14 items (seven worded positively) that measure perceived global stress and coping ability in the past month, on a scale from 0 = never to 4 = very often. This measure is commonly used, has high reported concurrent and predictive validity, adequate internal and test-retest reliability, and a relatively low participant burden. The questions are general in nature and hence relatively free of cultural bias.

#### Childhood Trauma Questionnaire (CTQ).

The CTQ is a 28-item, self-report inventory for participants aged 12 or older that taps five types of maltreatment: emotional, physical and sexual abuse, and emotional and physical neglect [133,134]. This questionnaire asks individuals to rate their experiences on a 5 point Likert scale (1 = never true to 5 = very often true).

#### Community Experiences Questionnaire (CEQ).

The CEQ is a 25-item self-report measure of individuals’ experiences of community violence, with two subscales to assess the frequency at which individuals were directly victimized by or witnessed community violence, ranging in severity from threats to killings [135,136].

#### Everyday Discrimination Scale (EDS).

The EDS is a 9-item self-report measure that is used to determine an individual’s subjective experiences of discrimination in their day-to-day lives [137]. Participants are asked to describe the frequency in which they have been exposed to each of these experiences on a 6-point Likert Scale: 0 = Never, 1 = Less than once a year, 2 = A few times a year, 3 = A few times a month, 4 = At least once a week, and 5 = Almost every day. If participants respond with “A few times a year” or more frequently to at least one question, they are then asked to report what they believe is the main reason for these experiences, e.g., your gender, your race, your age, your education or income level, your sexual orientation, etc. This measure has been validated in both a study focused on African American adults [138] and a broader study focused on racism and health [139].

#### Experiences of Discrimination (EOD).

The EOD questionnaire is a self-report measure of a number of constructs relating to discrimination, including experiences of situational discrimination, frequency of discriminatory experiences, response to discrimination, and worries about discrimination [139].

#### Multigroup Ethnic Identity Measure (MEIM).

The MEIM is a 12-item measure of membership in and identification with ethnic groups [140].

#### Vancouver Index of Acculturation (VIA).

The VIA [141] (Ryder, Alden & Paulhus, 2000) is a 20-item measure of acculturation that measures both the acquisition of the new cultural tendencies as well as the loss of old cultural tendencies.

#### PRIME with Distress and Attributions.

The PRIME Screen is a self-report measure of presence and severity of psychosis-risk/psychosis-like symptoms [141]. The measure contains 12 Likert-type items, with response options ranging from 0 (“definitely disagree”) to 6 (“definitely agree”), and has demonstrated adequate psychometric performance relative to clinician interview diagnoses of risk [142]. To capture distress associated with each symptom, a distress item was added to the Prime Screen for all items endorsed at a 1 (“somewhat disagree”) or higher. Additionally, at the baseline visit only, participants will be asked to give an example of a time they experienced a given symptom (example) and will be asked to list what they think caused this experience/symptom (attribution) to collect quasi-qualitative data on participants’ understanding of each Prime item.

#### Center for Epidemiologic Studies-Depression Scale (CES-D).

The CES-D [143] will be used to ascertain levels of depression. The original scale is a 20-item self-report scale designed to assess the presence and severity of depressive symptoms occurring over the past week in the general population. Respondents rate each item on a 4-point scale: 0 = rarely or none of the time, 1 = some or a little of the time, 2 = occasionally or a moderate amount of the time, and 3 = most or all of the time. Responses are summed to obtain total scores with higher scores indicative of high depressive symptoms, but not necessarily clinical depression. The present study will utilize a shortened version of the CES-D [144]. This shortened version includes 14 of the original 20 items that were grouped together based on factor analysis and load onto the three factors of negative affect, anhedonia, and somatic symptoms.

#### State Trait Anxiety Inventory Trait Form Anxiety Subscale Formatted [145].

This scale assesses symptoms of anxiety and worry. It consists of 40 items, which contain only the items from the STAI-trait form that loaded on the anxiety factor in the study by Bieling et al. [145] and excludes those items that loaded predominantly on the depression factor. The items are scored on a 4-point likert scale: 1 = Not at all, 2 = Somewhat, 3 = Moderately so, and 4 = Very much so. Although anxiety is related to increases in psychotic symptoms in schizophrenia populations, no study has determined whether anxiety is related to increases in minor psychotic symptoms in non-clinical samples, which will be determined in the present study.

#### Social Phobia Scale (SPS).

The SPS [146] was designed to assess anxiety symptoms related to performing various tasks (writing, drinking, eating in public) while being observed by other people. It consists of 20 items. Each item is rated on a 5-point scale that ranges from 0 (not at all characteristic or true of me) to 4 (extremely characteristic or true of me).

#### Life Events Checklist (LEC).

Items from the LEC [147], which was developed at the National Center for PTSD; will be used to determine exposure to potentially traumatic events. The LEC items requires respondents to indicate whether their experience of the event, on a 5-point nominal scale (1 = happened to me, 2 = witnessed it, 3 =learned about it, 4 = not sure, and 5 = does not apply). The events from the checklist include the following example items: Natural disaster, Accident, Combat, Death of loved one, Injury/illness of loved one, Witness family violence, Childhood physical assault, Adult physical assault, and Victim of bullying was added by the investigators.

#### Pittsburgh Sleep Quality Index (PSQI).

The PSQI [148] is a 10-item self-report questionnaire that evaluates sleeping habits in the past month. We include the PSQI because of accumulating data suggestive of a link between sleep disturbance and schizophrenia [149]. However, sleep disturbance in those with subthreshold psychotic symptoms has yet to be studied.

#### Motor and Activity Psychosis-Risk Scale (MAP-RS).

Dr. Vijay Mittal (PI) created the MAP-RS, a 17-item questionnaire that assesses aspects of motor-physical activity (e.g., clumsiness, balance) that have been found to be affected in some individuals who later develop psychosis [150].

#### Defeatist Performance Beliefs Scale (DPB).

The DPB [151] (Grant & Beck, 2009) is a 15-item, self-report measure used to evaluate the severity of defeatist performance beliefs. These are negative expectancies individuals sometimes have about performing goal-directed activities and socializing.

#### Treatment History Questionnaire (TRHQ).

The TRHQ assesses past and current experiences with mental health services including therapy, medications, diagnoses, and hospitalizations, as well as whether, to what degree, and for what type of mental health issues participants are considering seeking treatment. Our collaborator created this questionnaire and collected data from over 400 undergraduates at University of Maryland-Baltimore County (UMBC), with initial validity findings suggesting that students who reported current anxiety diagnoses had significantly elevated Beck Anxiety Index scores (means indicating “severe” anxiety) compared to non-endorsers and students who reported current depression diagnoses had significantly elevated BDI-II scores (means indicating “moderate” depression) compared to non-endorsers.

#### COVID-19 Questionnaire.

This questionnaire was developed to assess the effects of the COVID-19 pandemic on lifestyle and mental factors that can be used to gauge mental health outcomes associated with the pandemic.

#### Post-traumatic Stress Disorder Checklist-Civilian Version (PCL-C).

The PCL-C is a 17-item self-report instrument where items correspond to PTSD symptoms and ask the individual to report on how often certain symptoms were bothersome to them (1 = not bothersome at all through 5 = extremely bothersome) in the past month [152].

#### Drug Use Frequency Measure (DUF).

This questionnaire assesses drug and alcohol use within the past 3 months, as well as a quick assessment of health concerns and medication use [153]. The purpose of including this questionnaire in the present study is due to the known relationships between substance use and increased risk for schizophrenia and minor psychotic symptoms, as well as the high comorbidity between schizophrenia and substance use [7].

#### Edinburgh Handedness Inventory-Short Form.

This short-form, self-report measure will be used to assess handedness in participants and inform analysis of computerized task performance [154].

#### Puberty Development Scale.

Participants will be asked to respond to questions from the Puberty Development Scale to further supplement hormone data used in this study (level of development will be entered as a covariate). This scale is a 5-item scale rating on three measurements; physical development, an overall maturation measure, and a categorical measure [155].

#### Control Over Voice-hearing Experiences Scale.

This scale was developed to measure the degree and strategies that individuals with VHE can control their voices. The current scale measures the efficacy of exerting control over the VHE and two other dimensions associated with the strategies or means individuals use to exert control; the ability to manage either when the voices appear (direct control) or to use other factors to minimize how impactful or disruptive the voices are when they do appear (indirect control), or some combination thereof [156].

#### Revised Green et al. Paranoid Thoughts Scale (R-GPTS).

A self-report scale used to capture paranoia—the belief that others have bad intentions towards us—along the continuum from health to illness, and across diagnoses [157].

#### Auditory Hallucinations Rating Scale (AHRS).

This scale will be used to measure the hallucination state in participants who endorse auditory hallucinations [158].

#### Launay-Slade Hallucination Scale-Revised (LSHS-R).

The LSHS-R is a 12 item self-report questionnaire that measures predisposition to hallucinations in the general population using a five-point Likert Scale response format. Three subscales characterized as (a) vivid mental events, (b) hallucinations with a religious theme, and (c) auditory and visual hallucinatory experiences are part of the scale [159].

#### Peters’ Delusions Inventory (PDI-21).

The PDI-21 is a 21 item, dichotomous (Yes/No) self-report questionnaire to assess delusional symptoms in the general population. The higher the score, the greater the delusional symptoms. For each item, three follow up questions of 5 categories of response (1 to 5) are provided corresponding to the subscales of the degree of conviction, preoccupation, and distress [78,160].

## DATA ANALYSES, HYPOTHESES, AND PREDICTED OUTCOMES

Power estimates are based on a 20% conversion rate based on recent literature [161]. The analyses involving longitudinal time points (i.e., conversion, change in function) are estimated with the sample size of 300 per group (those with 24-month time points). We will also evaluate these aims at the 12-month point (*n* = 400 per group) in an exploratory fashion, as there is ample conversion at this point [7,12,17,21,121]. We use the 24-month time points data set to describe our methods that are also applicable to the 12-month time point data.

Machine learning (ML) and training/testing validation scheme: ML predictive models will be developed to calculate the probabilistic score of converting to psychosis and to predict change in functional outcome. We adopt ML models for the analysis because our goal is to predict outcomes (both binary and continuous) rather than demonstration of associations between an outcome (e.g., conversion) and test items. A repeated nested training-testing scheme will be used to avoid overfitting. Specifically, the 900 subjects (those with 24-month time points) collected in the first three years will first be randomly and proportionally divided into an outer-training set (*n*_r_ = 600) and an outer-testing set (*n*_s_ = 300). The 2:1 training and testing split ratio is used to achieve optimal performance. Within the outer-training set (*n*_r_ = 600), we will perform 5-fold repeated nested cross validation (CV) to optimize the model and tune parameter selection [162]. During 5-fold CV, 480 subjects will be used as the inner training set and the remaining 120 subjects as inner testing. The predictive model that achieves best averaged performance in 5-fold CV will be selected as the final model. In the testing stage (testing on the *n*_s_ = 300 subjects), the final model is locked, and the ML development team will be blinded to the outcome of these 300 subjects. Next, the outcome variables of psychosis conversion (binary) and functional outcome (continuous) will be calculated/predicted by the fixed ML models. The performance of the predictive models (comparing the predicted with true outcomes) will be evaluated based on the hold-out testing data set (*n*_s_ = 300) using metrics described below. The hypotheses in both aims will be assessed by the performance of predictive results.

### Aim 1A: To Develop a Psychosis-Risk Calculator through the Application of ML Methods to the Performance-Based and Self Report Data Generated by the CAPR Battery

We will perform comprehensive model evaluation using the outer-training set (*n*_r_ = 600). A variety of classifiers including regularized logistic regression, gradient boosting, and random forest among others will be considered as candidates [163]. The F1 score, which is the harmonic average of the true positive rate and positive predictive value, will be used as the model selection criteria in the training stage to account for the 20% conversion rate [164]. These predictive models along with the tuning parameters will be determined by 5-fold CV. We will also perform variable selection using regularization techniques (e.g., elastic net) to obtain a minimum set of features from all task measures and demographic variables including sex, age, race, and years of education. In addition, study site will be included as a variable in this analysis and, if systematic variance between sites emerges, adjustments to the model will be considered (e.g., quantile normalization). The tuning parameters of the regularization model are determined by the 5-fold CV. In general, the model with the minimum set of features is preferred if the evaluation metric of this model is no more than 3% lower than the model with optimum performance but using a larger set of features. The rationale is to reduce administration time while maintaining predictive accuracy.

The final model will be selected based on the training data set (*n*_r_ = 600), locked, and then applied to the hold- out testing data set (*n*_r_ = 300). We will test the primary hypothesis by performing an independent sample *t*-test between CHR converters and non-converters. In addition, we will use Monte Carlo-based tests to examine whether the F1 score > 0.75 because this nonparametric index is robust and does not require assumptions for asymptotic inference. We will also explore whether including self-report measures as predictors can improve the risk calculator performance. We will use a combination of linear and logistic regression to examine if comorbidity (CHR with mood or anxiety disorder/symptoms) impacts baseline performance on the CAPR calculator and whether it impacts predictive accuracy of the calculator. If it impacts accuracy, we will consider using co-morbid diagnosis as a potential predictor for ML analysis or using it as a covariate to adjust prediction scores of the ML model depending on which appears to be more appropriate. We will take the same analytic approach to medication type (antidepressant, anxiolytic, antipsychotic, stimulant). With a sample size of *n*_r_ = 300 (testing data set) and 100 CHR subjects, we would have power of 0.80 to detect a small effect size Cohen’s *d* = 0.31 (comparing converters vs non-converters) at the α = 0.05.

### Aim 1B: To Evaluate Group Differences on the Risk Calculator Score

Preliminary analyses will examine whether any of the following covariates should be included: years of education, age, sex, ethnicity/race, and medication variables. An ANOVA and survival analysis will be conducted to evaluate group differences on the CAPR battery risk calculator score. For the ANOVA, significant effects will be followed-up by Fisher’s protected *t*-tests to test group effects because simulations have shown that this approach provides adequate family-wise Type I error protection and has greater power than other corrections for Type I error [165]. The ANOVA will use baseline data from all 5 years and compare CHR, HSC, and HC. We hypothesize that the CHR group will have a significantly higher risk calculator score than either the HSC or HC (i.e., CHR > HSC and CHR > HC). The power of the contrast analysis for the ANOVA will be greater than .80 to detect a small effect of Cohen’s *d* = 0.26 (comparing CHR with HSC and/or HC) with a sample size of 900 subjects (all subjects excluding training *n*_r_ = 600) and adjusted α = 0.05. The corrected α = 0.05 was used. The survival analysis will utilize data from participants who have baseline, 12-month follow-up, and 24-month follow-up data. We hypothesize that the CHR converters will have a significantly higher risk calculator score than CHR non-converters, HSC, and HC. Based on previous guidelines, this survival analysis will be adequately powered (i.e., 0.80) to detect small-to-moderate differences between converters and other groups [166].

### Aim 1C: Evaluate How Baseline CAPR Performance Relates to Symptomatic Outcome 2 Years Later

Specifically, we intend to examine: (1) symptomatic change treated as a continuous variable (e.g., SIPS Positive Symptom and NSI-PR total scores) and (2) conversion to psychosis. We hypothesize that the CAPR calculator: (1) will predict symptom course and (2) that the differences observed between converters and non-converters will be larger on the CAPR calculator than on the NAPLS calculator.

A growth curve model will be used to test hypothesis 1, as this will permit using data from all time points and thus provide the most reliable estimate of change in symptoms. A linear model will be specified, which will allow for a test of overall model fit (i.e., df = 1) and an estimate of symptom change across two years. Age, sex, and other baseline clinical conditions will be included as covariates (note: both dichotomous diagnostic variables and continuous symptom counts will be used). Our sample size will provide adequate power (i.e., >0.80) to estimate individual differences in growth curves.

Regression analysis will be performed to test hypothesis 2 using score~Group + Calculator + Group × Calculator. Note that the outcome “score” is on a normalized scale (e.g., using quantile normalization) to ensure that the CAPR calculator score is comparable to the NAPLS calculator. We reject the null hypothesis if the interaction term “Group × Calculator” is significant with the correct direction. We will also perform a Monte Carlo-based nonparametric test to examine whether the F1 score of the CAPR calculator is higher than NAPLS calculator. The nonparametric test is used because the asymptotic inference of F1 score can be difficult and unreliable. With a sample size of *n*_r_ = 300 (testing data set), we would have power of 0.80 to detect a medium effect size *f*^2^ = 0.22 for the regression analysis at α = 0.05.

### Aim 2: Use ML Methods, as above, to Develop Calculators That Predict (2A) Social, and, (2B) Role Function Deterioration, Both Observed over Two Years

Because negative symptoms are known to be more strongly linked to functional outcome than positive symptoms, we predict that negative symptom mechanism tasks will be the strongest predictor of functional decline in both domains. We will perform comprehensive model evaluation using the outer-training set (*n*_r_ = 600). A variety of continuous outcome predictive models including regularized regression model (e.g., lasso, elastic net) gradient boosting, and random forest among many others will be considered. The *R*^*2*^ which evaluates how much variance of the change of the functional outcomes over 24 months can be explained by the ML-predicted outcome, will be used as the model selection criterion at the training stage and to evaluate the prediction accuracy at the testing stage [163]. We test the hypothesis by examining whether *r* = sqrt(*R*^*2*^) score > a medium effect size *r* = 0.30. Note that *r* value reflects the correlation between predicted and observed change in functional outcome. Power: With a sample size of *n*_r_ = 300 (testing data set), we would have power of 0.80 to reject the null hypothesis (*r* ≤ 0.30) at α = 0.05 when *r* is 0.42.

### Reproducibility

To ensure high scientific rigor and reproducibility, we will consider the effects of biological sex, assess and adjust inter-site difference as needed. In addition, we will perform model validation (e.g., using bootstrap techniques) to check the robustness, use multiple imputation for missing data (or full information maximum likelihood estimators, when appropriate), and sensitivity analysis to examine if data are missing at random. Specifically, multiple imputation will be implemented by multivariate imputation by chained equations in the “mice” package in *R* [167]. Last, we will make all of our code publicly available.

## POTENTIAL ISSUES, ALTERNATIVE APPROACHES, AND FUTURE DIRECTIONS

If we find that conversion rates are lower than anticipated, we will focus more on continuous measures (e.g., changes in symptoms and functioning over time) that are clinically important but are not contingent on transition rates. Relatedly, if a low conversion rate impacts the feasibility of the proposed statistical plan, we will explore supplementing the ML analyses with a psychometric approach (which will not rely on a proportion of converters being set aside as training set). As noted, Co-I Zinbarg has significant expertise with SEM methods, and will be actively involved in evolving our statistical strategy as unforeseen considerations may arise. Another issue arises if the NAPLS calculator outperforms the CAPR battery. Because the CAPR battery could be administered on the Internet, performance that roughly approximates NAPLS could have a large public health impact. However, the practical advantages of the CAPR approach are only relevant with a level of predictive accuracy that is sufficient to impact clinical practice. Beyond comparing calculators, we will also explore if the combination of the NAPLS and CAPR calculators provides greater accuracy than either calculator alone.

## SUMMARY OF CAPR STUDY

The CAPR study will develop and test a psychosis risk calculator based on machine learning techniques and a battery of computerized behavioral tasks, which are tied to the neurobiological systems and computational mechanisms implicated in psychosis. We believe that the CAPR battery and risk calculator have the potential to significantly improve the prediction of conversion to a psychotic disorder, through more closely assessing mechanisms involved in symptom expression and improving sensitivity relative to clinical interviewing methods. Additionally, given that these behavioral tasks can be administered online with limited expertise, we believe that the CAPR battery can expand access to psychosis risk assessment and thus have a significant public health impact.

## Figures and Tables

**Table 1.** CAPR Battery per Domain of Psychopathology.

Domain	Task	Time

Positive		

	Conditioned Hallucinations	40 min
	Kamin Blocking	18 min
	Probabilistic Reversal Learning Task	10 min
	Sine Wave Speech	11 min

Negative		

	Pessiglione	19 min
	Effort Expenditure for Rewards	24 min
	Delay Discounting	2 min
	Hedonic Reactivity	8 min
	Finger Tapping	27 min

Disorganized		

	Ebbinghaus Illusion	8 min
	Mooney Faces	4 min

Note: Tasks are described in the Preliminary studies section and the measures section.
